# Deletion of exon 4 in *LAMA2* is the most frequent mutation in Chinese patients with laminin α2-related muscular dystrophy

**DOI:** 10.1038/s41598-018-33098-3

**Published:** 2018-10-09

**Authors:** Lin Ge, Aijie Liu, Kai Gao, Renqian Du, Juan Ding, Bing Mao, Ying Hua, Xiaoli Zhang, Dandan Tan, Haipo Yang, Xiaona Fu, Yanbin Fan, Ling Zhang, Shujuan Song, Jian Wu, Feng Zhang, Yuwu Jiang, Xiru Wu, Hui Xiong

**Affiliations:** 1 Department of Pediatrics, Peking University First Hospital, Beijing, 100034 China; 2 Obstetrics and Gynecology Hospital, Institute of Reproduction and Development, Fudan University, Shanghai, 200011 China; 3 Department of Neurology, Wuhan Children’s Hospital, Wuhan, Hubei 430015 China; 4 Department of Pediatrics, Wuxi Children’s Hospital, Wuxi, Jiangsu 214023 China; 5 Department of Pediatrics, The Third Affiliated Hospital of Zhengzhou University, Zhengzhou, Henan 450052 China; 6 Department of Medical Genetics, School of Basic Medical Sciences, Peking University, Beijing, 100191 China; 7 MyGenostics, Inc., Beijing, 101318 China

## Abstract

Although recessive mutations in *LAMA2* are already known to cause laminin α2-related muscular dystrophy, a rare neuromuscular disorder, large deletions or duplications within this gene are not well-characterized. In this study, we applied next-generation sequencing-based copy number variation profiling in 114 individuals clinically diagnosed with laminin α2-related muscular dystrophy, including 96 who harboured *LAMA2* mutations and 34 who harboured intragenic rearrangements. In total, we detected 18 distinct *LAMA2* copy number variations that have been reported only among Chinese, 10 of which are novel. The frequency of CNVs in the cohort was 19.3%. Deletion of exon 4 was detected in 10 alleles of eight patients, accounting for 27% of all copy number variations. These patients are Han Chinese and were found to have the same haplotype and sequence at the breakpoint junction, suggesting that exon 4 deletion is a founder mutation in Chinese Han and a mutation hotspot. Moreover, the data highlight our approach, a modified next-generation sequencing assay, as a robust and sensitive tool to detect *LAMA2* variants; the assay identifies 85.7% of breakpoint junctions directly alongside sequence information. The method can be applied to clinical samples to determine causal variants underlying various Mendelian disorders.

## Introduction

Laminin α2-related muscular dystrophy (*LAMA2* MD) is a rare autosomal-recessive genetic disorder affecting between 0.7 and 2.5 in 100,000 individuals in predominantly European cohorts^1^. It is caused by pathogenic variants in *LAMA2* [MIM: 607855], which is located on chromosome 6q22-23 and consists of 65 exons^2^. Based on clinical features, it can be classified into two distinct entities, a severe, early-onset congenital muscular dystrophy (CMD), known as merosin deficiency or muscular dystrophy, congenital type 1A (MDC1A), which is the most frequent form of CMD, and the milder late childhood-onset limb girdle type muscular dystrophy (LGMD), known as LGMD R23 laminin α2-related^3^. Children with the severe form of the disease have profound hypotonia associated with muscle weakness at birth or during early infancy, poor spontaneous movements, joint contractures, and delayed motor milestones^4^. Unlike LGMD, these children usually do not gain independent ambulation. Specific abnormal cerebral white-matter signals are consistently observed by 1 year of age on T2-weighted MRI.

Currently, 553 unique sequence variants in *LAMA2* have been reported to the Leiden Open Variation Database (accessed June 2018). Pathogenic changes include small deletions and insertions, nonsense mutations, splice site mutations, and missense substitutions. However, few large deletions or duplications have been reported^5–7^. A suspected large *LAMA2* deletion, likely spanning exons 23 to 56, was initially identified by Pegoraro *et al*.^8^ bases on protein truncation test. Notably, the first fully characterized large deletion in *LAMA2*, a frameshift (out-of-frame) deletion of exon 56, was subsequently proven to be one of the most frequent pathogenic variants in Portuguese patients with MDC1A^9^. Similarly, Xiong *et al*.^7^ detected seven deletions of one or more exons in 43 Chinese patients. However, the copy-number variations (CNVs) spectrum and the characteristics of these CNVs have not been evaluated.

Genomic CNVs represent a major source of genetic diversity^10^. In the past decade, microarray-based profiling was introduced as a first-tier diagnostic test for genomic disorders and other diseases related to CNVs^11,12^. Additionally, multiplex ligation-dependent probe amplification (MLPA) enables the detection of many large deletions and duplications. However, these methods do not provide a comprehensive overview of CNVs in terms of breakpoint junctions, preventing full understanding of the pathogenic and mutational mechanisms. Although previous studies have highlighted the significance of CNVs in *LAMA2* MD, diagnostic genetic testing strategies are mostly targeted at small genetic variants using, for example, next-generation sequencing (NGS)^13^. In addition to array-based comparative genomic hybridization (aCGH), NGS approaches can be used to detect large structural variants. Unfortunately, accurate identification of CNVs at the nucleotide sequence level by NGS remains challenging^14^, even though several algorithms have been developed to detect CNVs in exomes^15–18^ and DNA samples^19,20^ based on depth-of-coverage.

The aim of this study was to (1) describe the spectrum of pathogenic deletions and duplications in *LAMA2* in a large cohort of patients with non-recurrent genomic rearrangements and (2) develop a modified NGS approach for *LAMA2* variant detection and identification. We hope to provide a *LAMA2* copy-number mutation spectrum and a diagnostic strategy for *LAMA2* genetic analyses.

## Materials and Methods

### Editorial policies and ethical considerations

The study was reviewed and approved by the Ethics Committee of Peking University First Hospital (No. 2015[916], Beijing, China). All patients and/or their parents provided written informed consent to participate in the study and granted permission to publish medical data. Methods were compliant with the relevant guidelines and regulations.

### Patient enrolment and analysis of *LAMA2* mutations

In 2004–2017, 114 patients were diagnosed with *LAMA2* MD at our institution. The inclusion criteria were a clinical diagnosis of muscular dystrophy characterized by muscle weakness or hypotonia with an early onset, delayed motor developmental milestones, motor-unit disease signs, a high creatine kinase level, and changes in brain white matter signals without typical structural changes observed in α-dystroglycanopathy or clinically diagnosed LGMD with typical white matter changes. Point mutations were detected by sequencing the *LAMA2* gene, including all coding and flanking intronic sequences. Combined with a dosage analysis by MLPA (SALSA MLPA Kit P391-A1/P392-A1; MRC-Holland, Amsterdam, the Netherlands) and aCGH, 96 individuals were found to harbour *LAMA2* mutations, including intragenic rearrangements in 34 patients from 29 families. Sufficient material for further studies was available from 29 probands, including 28 patients with deletions and 1 with a duplication. Detailed phenotypic data, including motor development, mental development, pattern of muscle involvement, joint contracture, serum creatine kinase level, brain MRI, and electromyography, obtained from all probands are listed in Table 1. DNA samples from patients and their parents were obtained from peripheral blood using the DNeasy Blood and Tissue Kit (Qiagen, Hilden, Germany).

**Table 1:** Clinical findings of 29 probands with *LAMA2* MD.

Subject	Sex	Age of onset	Age last seen/Max motor milestone	Highest serum CK U/I (age)	Scoliosis	Contracture	EMG myopathic changes	intellect/seizures	MRI (T2-weighted images)	IH staining of Laminin-a2
P1	F	Birth	Died when 9 years/sitting	1630 (17 months)	−	Yes	+Motor nerve CMAP amplitude reduced	Normal intellect/no seizure	WMH	n/a
P2	M	Birth	Died when 5 months/can’t sit	4100 (2 days)	−	Yes	+	Normal intellect/no seizure	n/a	n/a
P3	F	4 months	5 years/walking	2427 (9 months)	−	Yes	+	Normal intellect/no seizure	WMH	n/a
P4	M	Birth	5 years/sitting	1640 (11months)	−	Yes	n/a	Normal intellect/no seizure	WMH	−
P5	M	Birth	2 years/can’t sit	2312 (3 months)	−	Yes	+	Normal intellect/no seizure	WMH	n/a
P6	M	Birth	3 years/sitting	3148 (10 months)	−	Yes	n/a	Normal intellect/no seizure	WMH	n/a
P7	F	Birth	7 years/sitting	3264 (10 months)	+	Yes	+CMAP amplitude reduced	Normal intellect/no seizure	WMH	−
P8	M	Birth	Died when 9 years/sitting	91 (6 years)	+	Yes	+Motor NCS reduced	Normal intellect/no seizure	WMH	n/a
P9	M	Birth	5 years/sitting	2481 (8 months)	−	Yes	+Motor nerve CMAP amplitude reduced	Normal intellect/no seizure	WMH	n/a
P10	M	Birth	4 years/sitting	1778 (1 years)	−	Yes	+	Normal intellect/no seizure	WMH	−
P11	M	Birth	12 years/sitting	n/a	+	Yes	+	Intellectual decay/seizure	WMH	n/a
P12	F	Birth	3 years/sitting	1715 (11 months)	−	Yes	+	Normal intellect/no seizure	WMH	n/a
P13	F	6 months	3 year/walking	1337 (24 months)	−	Yes	+Motor NCS reduced	Normal intellect/no seizure	WMH	n/a
P14	F	Birth	5 months/can’t sit	8010 (2 months)	−	Yes	n/a	Normal intellect/no seizure	normal (when 5 months)	n/a
P15	M	Birth	6 years/sitting	491 (6 years)	+	Yes	+	Normal intellect/no seizure	WMH	n/a
P16	M	Birth	5 years/sitting	611 (2 years)	−	Yes	+	Normal intellect/no seizure	WMH	n/a
P17	M	Birth	6 years/sitting	2612 (3 months)	+	Yes	+	Normal intellect/no seizure	WMH	n/a
P18	F	Birth	8 months/can’t sit	3551 (8 months)	−	Yes	n/a	Normal intellect/no seizure	WMH	−
P19	M	Birth	2 years/sitting	2151 (5 months)	−	Yes	+	Normal intellect/no seizure	WMH	n/a
P20	M	Birth	3 years/sitting	3983 (3 months)	−	Yes	+	Normal intellect/no seizure	WMH	−
P21	M	7 years	9 years/running	2103 (6 years)	−	No	n/a	Normal intellect/no seizure	WMH	−
P22	F	4 months	10 years/walking	1565 (16 months)	+	Yes	+	Normal intellect/febrile seizure at 4 years	WMH	n/a
P23	F	Birth	1 years/can’t sit	14616 (2 days)	−	Yes	+	Normal intellect/no seizure	WMH	n/a
P24	M	Birth	1 years/sitting	3496 (16 months)	−	Yes	+	Normal intellect/no seizure	WMH	n/a
P25	F	Birth	2 years/sitting	2757 (6 months)	−	Yes	n/a	Normal intellect/no seizure	WMH	n/a
P26	M	Birth	10 years/sitting	3549 (6 months)	+	Yes	+	Normal intellect/no seizure	WMH	−
P27	F	Birth	2 years/sitting	4224 (8 months)	−	Yes	+	Intellectual decay/no seizure	WMH	n/a
P28	F	2 years	3 years/running	3078 (2 years)	−	No	+Motor NCS reduced	Normal intellect/no seizure	WMH	n/a
P29	F	Birth	5 years/sitting	1500 (2 years)	−	Yes	+	Normal intellect/no seizure	WMH	n/a

F, female; M, male; CK, creatine kinase; EMG, electromyography; CMAP, compound muscle action potential; NCS, nerve conduction speed; WMH, abnormal white matter hyperintensities on T2-MRI; IH, immunohistochemical; n/a, not available.

### Histochemistry and immunohistochemistry

Muscle biopsies were collected with informed consent from the biceps brachiis of P4, P7, P10, P18, P20, P21, and P26. Tissues were precooled with isopentane before fixation in liquid nitrogen. The histochemical staining and immunohistochemical staining were carried out and observed independently by two investigators. Laminin α2 chain was stained using 100 μL of the mouse monoclonal antibody MAB 1922 (1:5,000, 5H2, Merck Millipore, Darmstadt, Germany), and the C-terminus of dystrophin was stained with 100 μL of the mouse monoclonal antibody NCL-DYS2 (1:20, Dy8/6C5, Leica Biosystems, Newcastle, United Kingdom)^6^.

### High-resolution aCGH analysis

High-resolution *LAMA2*-targeted aCGH microarrays (SurePrint G3 Microarray, 4*180K) with average probe spacing 500 base pairs were synthesized to map *LAMA2* and 150 kb flanking regions. Probes were designed using the Agilent Technologies eArray tool (Santa Clara, CA, USA), and samples were tested according to the manufacturer’s recommendations. Data were analysed in Agilent Genomic Workbench version 7.0.

### NGS and accurate characterization of CNVs using a composite pipeline method

Genomic DNA samples were fragmented and prepared for standard Illumina library construction. Biotinylated capture probes (MyGenostics, Beijing, China) were designed along the entire region of the *LAMA2* gene (Hg19: chr6: 129204285–129837710) and sequenced using the Illumina HiSeq X Ten sequencer to obtain paired-end reads of 150 bp. Clean reads were mapped to the UCSC hg19 human reference genome using BWA. Single nucleotide polymorphisms and insertions/deletions were detected using HaplotypeCaller in GATK and functionally annotated using ANNOVAR against 1000 Genomes Project, ESP6500, dbSNP, ExAC, HGMD, and an in-house database. The pathogenicity of novel missense variants was scored using Polyphen-2, SIFT, and MutationTaster.

To predict breakpoints, structural variants were analysed in CREST according to Wang *et al*.^21^. Briefly, the soft-clipped reads were extracted from the binary alignment files, and putative breakpoints were assembled into a contig. The contig was then mapped against the reference genome (NM_000426.3) to identify candidate partner breakpoints and a match to the initial breakpoint was considered to indicate a structural variant.

Putative CNVs were identified by read-depth analysis, which is based on the ratio of reads in a test sample to reads in a control sample (Human Reference DNA mix, Promega, Madison, WI, USA). In particular, ratios less than 0.75 and above 1.25 were considered to indicate a potential deletion and duplication, respectively. CNVs of the full *LAMA2* gene and its exons were calculated, and analyzed to identify approximate breakpoint positions. Finally, precise breakpoints were mapped a second time from binary alignment files in the context of breakpoints predicted in CREST.

### Breakpoint-spanning long-range PCR and haplotype analysis

Long-range PCR and Sanger sequencing were performed to verify the parental derivation, and to determine sequences spanning breakpoints, as well as approximately 1,000 bp flanking each end. Primers were designed using Oligo 6.0. To analyse *LAMA2* duplication by NGS, primers were designed based on the predicted tandem duplication and on head-to-tail rearrangement^22^. To analyse *LAMA2* deletions by NGS and aCGH, primers were designed to amplify unique breakpoint junctions. Primer sequences are listed in Supplementary Table S1. Long-range PCR was performed using Takara PrimeSTAR GXL DNA Polymerase (Takara, Osaka, Japan). Variants were described according to Human Genome Variation Society guidelines for mutation nomenclature (version 2.0) and using the cDNA reference sequence (accession number NM_000426.3). Haplotype analysis was performed for P12, P13, and P14, who were heterozygous for deletion of exon 4 as previously reported^7^.

### Analysis of mutational mechanisms underlying CNVs

RepeatMasker was used to evaluate interspersed repeat-elements at breakpoint junctions, including short interspersed nuclear elements, long interspersed nuclear elements (LINEs), long terminal repeats, DNA repeat elements, and low-complexity repeats. BLAT was used to determine the origin of sequences inserted at junctions. Blunt ends at breakpoint junctions were considered to indicate non-homologous end-joining, while microhomology was considered to indicate microhomology-mediated break-induced replication or non-homologous end-joining. Rearrangements due to *Alu* and long interspersed nuclear elements were identified based on the presence of such elements at breakpoint ends.

## Results

### Patient characteristics

**Figure 1:**
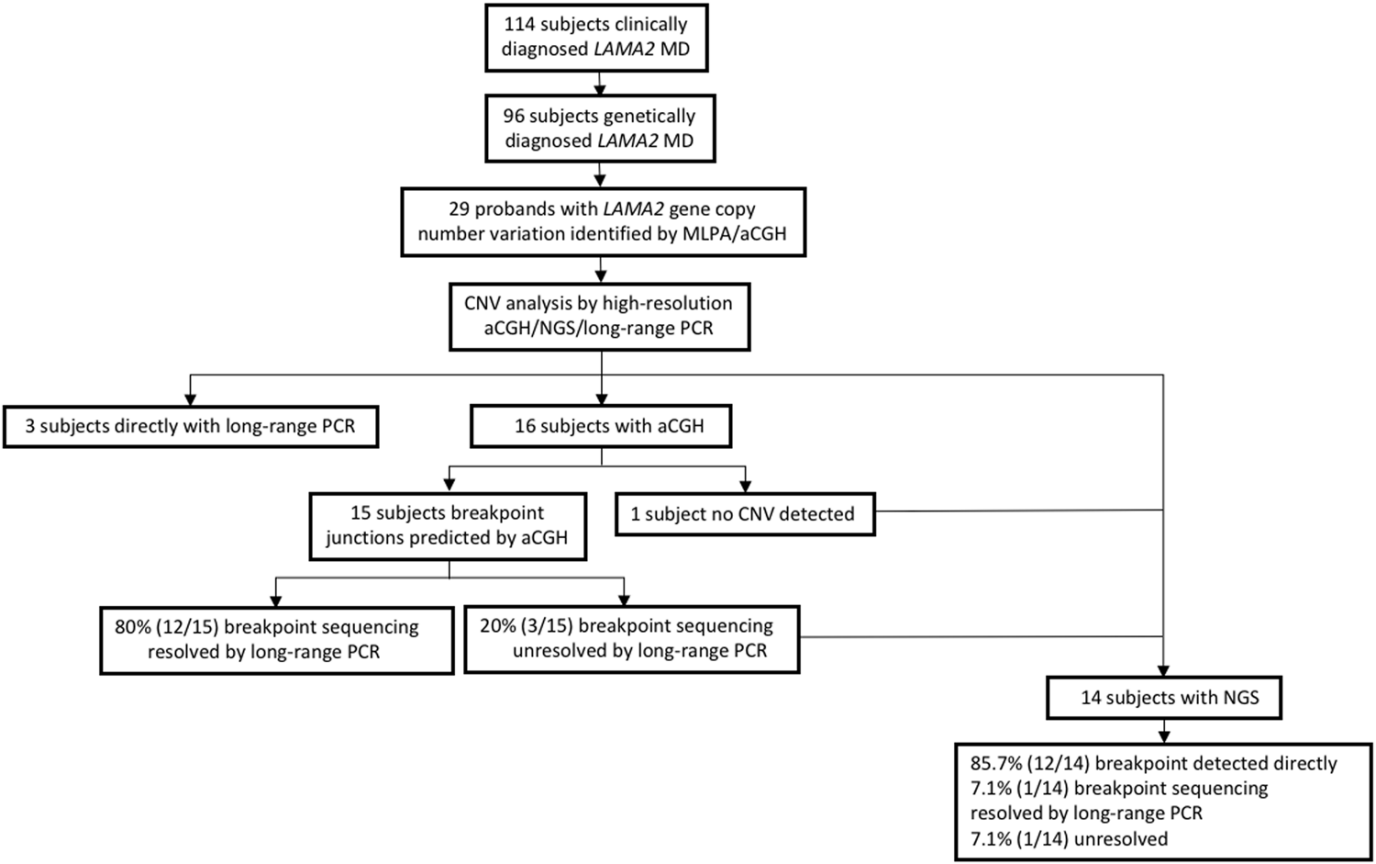
Workflow of CNV analyses and breakpoint sequencing for subjects with *LAMA2* MD-associated *LAMA2* gene CNVs. *LAMA2* gene CNVs were identified initially by MLPA assay and were further verified by high-resolution aCGH and next-generation sequencing. The sequence-based CNV structures were investigated comprehensively by CNV breakpoint sequencing.

The cohort was analyzed according to Fig. 1. Clinical and neuroradiological findings are listed in Table 1 for the 29 probands with *LAMA2* CNVs, of whom 27 were diagnosed with MDC1A and two were diagnosed with LGMD R23 laminin α2-related. Twenty-four probands had hypotonia and weak cry with onset at birth, while three had the same features and delayed milestones during the first 6 months. Furthermore, 24 probands never achieved independent ambulation, while five had mild muscle weakness with preserved ambulation capacity. Indeed, 13 of 96 patients genetically diagnosed with *LAMA2* MD (Fig. 1) were ambulant. Three probands died of severe pneumonia at 9 years and 5 months of age. Twenty-five probands presented moderately to significantly increased creatine kinase before 2 years, which decreased after the age of 3 years and returned to physiological levels until follow-up at 6 years and older. Brain MRI showed bilateral alterations in T2 intensities in periventricular white matter after 6 months in all probands, with sparing of the corpus callosum, internal capsule, cerebellum, and brain stem. Diffuse white matter abnormalities were observed in four cases, possibly resulting in comorbid mental retardation (two patients) and epileptic seizures (two patients). Electromyography revealed myogenic damage in 23 probands, and 6 patients showed mild abnormality of peripheral nerve compound muscle action potential and nerve conduction speed. Haematoxylin and eosin staining of seven muscle biopsies showed considerable proliferation of connective and fat tissue and substantial variability in the size of muscle fibres. As assessed by immunohistochemistry of the seven biopsies, laminin α2 was weakly expressed in the one mild case (P21), but absent from the six typical cases (P4, P7, P10, P18, P20, and P26, Table 1).

### Point mutations and novel CNVs in *LAMA2*

Six novel point mutations that were detected are listed in Table 2, including one nonsense mutation (c.6433A > T, p.K2145*), two frameshift mutations (c.2526_2529insACGC, p.C844Tfs*3 and c.3146del, p.G1050Afs*25), one mutation at a splice site (c.3038-7G > A), and two missense mutations (c.6584T > G, p.L2195P and c.8906G > C, p.R2969P). The missense and splice site mutations were predicted to be likely pathogenic and of uncertain significance, respectively, according to American College of Medical Genetics and Genomics classification^23^. Further characterization by immunohistochemistry showed complete laminin-α2 deficiency in all three patient (P4, P18, and P20).

**Table 2:** Information of the *LAMA2* mutation analysis.

Subject	LAMA2 exon/intron	Nucleotide change	Predicted amino acid change	Novel/reported	Parental derivation	Methods Used to Obtain the Predicted Breakpoint Junctions in Unique Regions		
						NGS	aCGH	Long-range PCR
P1	Out-of-frame deletion 50	Exon1del c.7147C > T	p.R2383*	Novel Reported^38^	Maternal Paternal	NA	S	NA
P2	Out-of-frame deletion 56	Exon1del c.7810C > T	p.R2604*	Novel Reported^7^	Maternal Paternal	S	NA	NA
P3	Out-of-frame deletion IVS35	Exon2-3del c.5071 + 1G > A		Reported^7^ Reported^7^	Paternal Maternal	NA	S	NA
P4	In-frame deletion 47	Exon2-9del c.6584T > C	p.L2195P	Novel Novel	Paternal Maternal	NA	S	NA
P5	Out-of-frame deletion 63	Exon2-12del c.8906G > C	p.R2969P	Novel Novel	Maternal Paternal	S	NA	NA
P6	Out-of-frame deletion Out-of-frame deletion	Exon3-4del Exon3-4del		Reported^7^ Reported^7^	Maternal Paternal	NA	S	NA
P7	In-frame deletion 27	Exon4del c.3955C > T	p.R1319*	Reported^7^ Reported^7^	Paternal Maternal	NA	S	NA
P8	In-frame deletion In-frame deletion	Exon4del Exon4del		Reported^7^ Reported^7^	Paternal Maternal	NA	S	NA
P9	In-frame deletion In-frame deletion	Exon4del Exon4del		Reported^7^ Reported^7^	Paternal Maternal	NA	S	NA
P10	In-frame deletion 19	Exon4del c.2565delC	p.S856Lfs*32	Reported^7^ Reported^7^	Paternal Maternal	NA	S	NA
P11	In-frame deletion 57	Exon4del c.7921G > T	p.E2641*	Reported^7^ Reported^7^	Paternal Maternal	NA	S	NA
P12	In-frame deletion 50	Exon4del c.7147C > T	p.R2383*	Reported^7^ Reported^38^	Paternal Maternal	NA	NA	S
P13	In-frame deletion 6	Exon4del c.830C > T	p.S277L	Reported^7^ Reported^39^	Maternal Paternal	NA	NA	S
P14	In-frame deletion 50	Exon4del c.7147C > T	p.R2383*	Reported^7^ Reported^38^	Paternal Maternal	NA	NA	S
P15	In-frame deletion 14	Exon5del c.2049_2050delAG	p.R683Sfs*21	Reported^7^ Reported^40^	Maternal Paternal	NA	S	NA
P16	In-frame duplication 37	Exon5-8dup c.5290_5291insG	p.E1764Gfs*3	Novel Reported^41^	Maternal Paternal	S	NA	NA
P17	Out-of-frame deletion 64	Exon10-12del c.9101_9104dup	p.H3035Qfs*5	Reported^7^ Reported^8^	Paternal Maternal	S	F	NA
P18	Out-of-frame deletion IVS21	Exon13-14del c.3038-7G > A		Novel Novel	Maternal Paternal	S	NA	NA
P19	Out-of-frame deletion 46	Exon20del c.6466C > T	p.R2156*	Novel Reported^42^	Paternal Maternal	S	NA	NA
P20	Out-of-frame deletion 63	Exon30del c.8906G > C	p.R2969P	Novel Novel	Maternal Paternal	S	NA	NA
P21	Out-of-frame deletion 10	Exon36-65del/c.1358G > C	p.C453S	Reported^6^ Reported^6^	Paternal Maternal	F	F	NA
P22	Out-of-frame deletion 4	Exon41-47del c.482_485dup	p.E162Dfs*1	Reported^7^ Reported^7^	Maternal Paternal	NA	S	NA
P23	Out-of-frame deletion 18	Exon49del c.2526_2529insACGC	p.C844Tfs*3	Novel Novel	Maternal Paternal	S	F	NA
P24	Out-of-frame deletion 50	Exon49del c.7174C > T	p.R2383*	Novel Reported^38^	Paternal Maternal	S	NA	NA
P25	Out-of-frame deletion 46	Exon49-57del c.6433A > T	p.K2145*	Novel Novel	Paternal Maternal	S	NA	NA
P26	In-frame deletion 3	Exon59-63del c.363C > G	p.Y121*	Reported^7^ Reported^7^	Paternal Maternal	NA	S	NA
P27	In-frame deletion 63	Exon59-63del c.8906G > C	p.R2969P	Reported^7^ Novel	Paternal Maternal	S	NA	NA
P28	In-frame deletion 22	Exon59-63del c.3146del	p.G1050Afs*25	Reported^7^ Novel	Maternal Paternal	S	NA	NA
P29	Out-of-frame deletion 27	c.8910_8965del c.4048C > T	p.T2921Yfs*2 p.R1350*	Novel Reported^43^	Maternal Paternal	S	F	NA

Abbreviation: F, fail; NA, not applied; S, succeed.

Previously, we reported seven large deletions encompassing one or more *LAMA2* exons in 43 patients with *LAMA2* MD^7^, indicating that CNVs are relatively frequent among our clinical patients, at approximately 20%. Of the 96 cases genetically diagnosed with *LAMA2* MD, 34 (35.4%) harboured heterozygous or homozygous intragenic rearrangements, such that the overall frequency of *LAMA2* CNVs was 19.3% (37/192 alleles). Eight patients of Han descent (P7–P14) were homozygous or heterozygous for deletion of exon 4 from a total of 10 alleles. AACAA microhomology was observed at breakpoints in all 10 alleles, and further haplotype analysis identified a founder mutation corresponding to an in-frame deletion of 5,465 bp. Finally, immunohistochemistry showed complete laminin-α2 deficiency in P4, P7, and P10.

Heterozygous and homozygous CNVs were detected in 26 and 3 probands, respectively, and confirmed by analyzing the patients’ parents (Table 2). We identified ten novel CNVs, including nine deletions and one duplication, most (8/10, 80%) of which were predicted to cause frameshift (out-of-frame deletions). As illustrated in Fig. 1, long-range PCR was used to directly investigate six putative breakpoint junctions in three cases with exon 4 deletions. High-resolution aCGH was used to investigate 16 cases with *LAMA2* MD (Supplementary. Fig. S1). Among them, 14/16 (87.5%) patients with simple CNV patterns, indicating simple genomic rearrangements, were observed. High-resolution aCGH also revealed potential CNV structural complexity in P22 (DEL-NML-DEL) that was not detected using the low-resolution MLPA. CNV was not detected by aCGH in the remaining case (P29), even though deletion of exon 63 was detected by MLPA. Further long-range PCR was used to resolve the CNV base pairs, allowing the amplification of 24/30 (80%) putative CNV breakpoint junctions. Notably, further long-range PCR was used to evaluate P17 as both junction ends overlapped with repeat elements. NGS was subsequently used to investigate the remaining 10 cases who were not analysed by aCGH, along with three cases (P17, P21, and P23) with no amplification product by long-range PCR and the one case (P29) in whom CNV was detected only by MLPA (Supplementary Fig. S2 and Table S2). Remapping resulted in the identification of a 56 bp deletion in exon 63 in P29. Of the three cases that were not resolved by long-range PCR, NGS was used to successfully resolve two, with the exception of P21, which was too large for the *LAMA2*-target NGS analysis coverage used in this study. All 10 cases where aCGH was not used were resolved directly using NGS data. Overall, NGS allowed direct identification of 12/14 (85.7%) putative CNV breakpoint junctions at CNVs.

### Breakpoint characteristics and CNV mutational mechanisms

**Figure 2:**
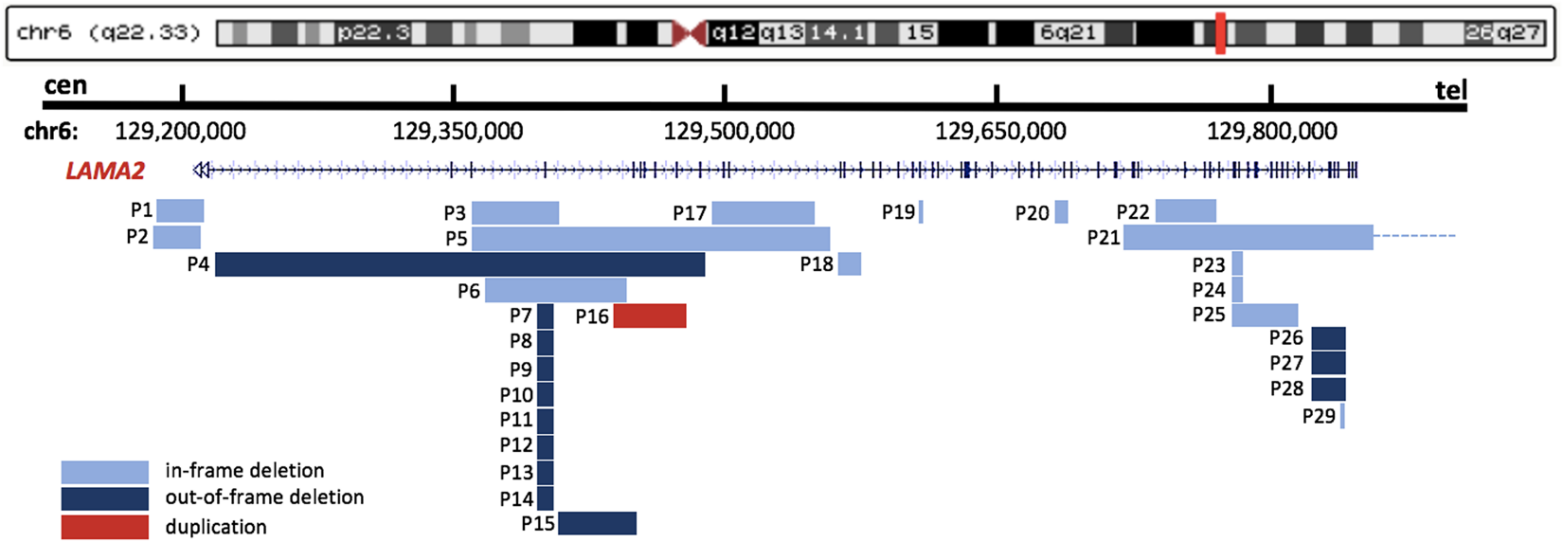
Global View of Identified *LAMA2* Intragenic CNVs. The genomic structure of *LAMA2* is presented in UCSC Genome Browser GRCh37/hg19, and custom tracks show *LAMA2* intragenic CNVs. In-frame deletions were annotated with light blue color, out-of-frame deletions were annotated with dark blue color, and duplications were highlighted in red color. CNV in P21 was large and exceeded the coverage of the *LAMA2*-target aCGH utilized in this study, the dotted line was used to indicate the location of the uncertain breakpoint downstream.

*LAMA2* intragenic rearrangements are dispersed throughout the gene, varying in size, location, and rearrangement mechanisms (Fig. 2). Rearrangement sizes ranged from 1.3 kb to 267.1 kb, with average 71.1 kb and median 14.3 kb (Supplementary Fig. S3). Genomic rearrangements span 1 to 29 protein-coding exons, although the distribution was biased towards smaller rearrangements. In total, 71.4% (20/28) of CNVs were in the N-terminal domain (exons 1–30), especially exons 3–4. Other CNVs were found in the G domain (exons 46–63) at the C-terminus, which are believed to disrupt of the link between the extracellular matrix and dystrophin-glycoprotein^24^.

Sequencing and alignment to the UCSC hg19 human reference genome of breakpoints at all 26 deletions (except that in P21) and 1 duplication (Table 3 and Supplementary Fig. S4) revealed simple non-recurrent rearrangements with microhomology at breakpoint junctions in 20/27 (74.0%) individuals, suggesting double-strand DNA breaks followed by microhomology-mediated end-joining. In 5/27 (18.5%) rearrangements, an insertion of 1–328 bp was found between breakpoints. One of these insertions (in P22) was most likely mediated by fork stalling and template switching/microhomology-mediated break-induced replication, and one, a short stretch of 28 bp in P6, was mapped to the reference sequence close to the breakpoint, suggesting serial replication stalling and re-replication. CNVs in P4, P23, and P24 contained short insertions with random nucleotides (<5 bp) at breakpoints, suggesting replication-independent non-homologous end-joining. Only breakpoint junctions in P5 and P25 consisted of two blunt ends without insertions. Finally, the duplication was confirmed to be a tandem duplication at the *LAMA2* locus.

**Table 3:** Breakpoint Characteristics of CNVs.

Subject	CNV Structure	Brekpoint Coordinate (GRCh37/hg19)	Length (bp)	Breakpoint Sequence Characteristics				Preferred Mutational Mechanism	Error Near Breakpoint (chr6)
				Homologous Genomic Repeats	Microhomology	Insertion	Blunt Ends		
P1	DEL	[Chr6:129187644]:[Chr6:129212825]	25,181 bp	No	CA	No	No	NHEJ or MMBIR	No
P2	DEL	[Chr6:129185304]:[Chr6:129210133]	24,829 bp	No	TCTT	No	No	NHEJ or MMBIR	No
P3	DEL	[Chr6:129355433]:[Chr6:129402434]	47,001 bp	No	TAA	No	No	NHEJ or MMBIR	G > T at 129355411; C > T at 129402537
P4	DEL	[Chr6:129221788]:[Chr6:129488858]	267,070 bp	No	No	A	No	NHEJ	C > G at 129488876; T > A at 129488886
P5	DEL	[Chr6:129354701]:[Chr6:129548523]	193,822 bp	No	No	No	Yes	NHEJ	No
P6	DEL	[Chr6:129379289]:[Chr6:129454975]	75,686 bp	No	No	TCGTAAAATACACA CACACACACACTCC	No	NHEJ	A > C at 129378992
P7	DEL	[Chr6:129414981]:[Chr6:129420437]	5,465 bp	No	AACAA	No	No	NHEJ or MMBIR	No
P8	DEL	[Chr6:129414981]:[Chr6:129420437]	5,465 bp	No	AACAA	No	No	NHEJ or MMBIR	No
P9	DEL	[Chr6:129414981]:[Chr6:129420437]	5,465 bp	No	AACAA	No	No	NHEJ or MMBIR	No
P10	DEL	[Chr6:129414981]:[Chr6:129420437]	5,465 bp	No	AACAA	No	No	NHEJ or MMBIR	No
P11	DEL	[Chr6:129414981]:[Chr6:129420437]	5,465 bp	No	AACAA	No	No	NHEJ or MMBIR	No
P12	DEL	[Chr6:129414981]:[Chr6:129420437]	5,465 bp	No	AACAA	No	No	NHEJ or MMBIR	No
P13	DEL	[Chr6:129414981]:[Chr6:129420437]	5,465 bp	No	AACAA	No	No	NHEJ or MMBIR	No
P14	DEL	[Chr6:129414981]:[Chr6:129420437]	5,465 bp	No	AACAA	No	No	NHEJ or MMBIR	No
P15	DEL	[Chr6:129423549]:[Chr6:129465235]	41,686 bp	No	GAT	No	No	NHEJ or MMBIR	A inserted at 129423453–129423454
P16	DUP	[Chr6:129440069]:[Chr6:129478884]	38,815 bp	No	A	No	No	NHEJ or MMBIR	No
P17	DEL	[Chr6:129488856]:[Chr6:129544038]	55,182 bp	L1PA2:L1PA5	No	No	No	LINE-mediated rearrangement	C > T at 129544095
P18	DEL	[Chr6:129566786]:[Chr6:129578227]	11,441 bp	No	A	No	No	NHEJ or MMBIR	A > G at 129578335
P19	DEL	[Chr6:129612189]:[Chr6:129613535]	1,346 bp	No	A	No	No	NHEJ or MMBIR	A inserted at 129613597–129613598
P20	DEL	[Chr6:129658427]:[Chr6:129664812]	6,390 bp	No	AGTACA	No	No	NHEJ or MMBIR	No
P21	DEL	[Chr6:129710417–129711135]- [Chr6:129837710+]	NA	NA	NA	NA	NA	NA	NA
P22	DEL-NML-DEL	[Chr6:129746506]:[Chr6:129775772] [Chr6:129776069]:[Chr6:129779042]	29,266 bp 2,973 bp	No	GC AC	ACCCAAAACTCCCTGTTAAACCCAAAACAGC	No	NHEJ or MMBIR	No
P23	DEL	[Chr6:129778345]:[Chr6:129782637]	4,292p	No	No	TA	No	NHEJ	No
P24	DEL	[Chr6:129778345]:[Chr6:129782637]	4,292p	No	No	TA	No	NHEJ	No
P25	DEL	[Chr6:129778271]:[Chr6:129813338]	35,067 bp	No	No	No	Yes	NHEJ	C > G at 129778200; T > C at 129813347
P26	DEL	[Chr6:129816374]:[Chr6:129833601]	17,227 bp	No	CAAA	No	No	NHEJ or MMBIR	No
P27	DEL	[Chr6:129816374]:[Chr6:129833601]	17,227 bp	No	CAAA	No	No	NHEJ or MMBIR	No
P28	DEL	[Chr6:129816374]:[Chr6:129833601]	17,227 bp	No	CAAA	No	No	NHEJ or MMBIR	No
P29	DEL	[Chr6:129833559]:[Chr6:129833615]	56 bp	—	—	—	—	—	—

DEL, deletion; DUP, duplication.

Using RepeatMasker in the UCSC Genome Browser, 96 *Alu* and 83 partial L1 repetitive elements longer than 100 bp were detected throughout the *LAMA2* non-coding region (Supplementary Fig. S5). Indeed, 11 of 27 (40.7%) junction ends overlapped with at least one type of repeat element, which is much lower than in the *DMD* gene^25,26^, but higher than the average frequency (30.5%) of repetitive sequences in *LAMA2*. Only the rearrangement in P17 is likely to be due to L1-mediated non-allelic homologous recombination, with a 471 bp region of identity. *Alu* elements were not involved in genomic rearrangements in our cohort. A high frequency of single- or oligonucleotide changes close to the breakpoints was detected in the *LAMA2*-associated CNVs analyzed in this study (Table 2), all of which are absent from 1000 Genomes Project and ExAC^27^, consistent with error-prone replicative repair mechanism in CNV mutagenesis^28^.

### Correlation between CNV contents and disease severity

The length of deletions and the corresponding exons that are deleted may partially account for the spectrum of *LAMA2* MD phenotypes and severity. P29, who does not harbour CNVs, was excluded from the following analysis of genotype-phenotype correlation. The remaining 28 probands were categorized into two groups according to phenotype. In a comparison of *LAMA2*-associated CNV sizes, the deleted genomic segments were considerably longer in the LGMD R23 laminin α2-related group (P21, P28) than in the MDC1A group. Both deletions in the LGMD R23 laminin α2-related group were located in the G domain at the C-terminus. One half of the *LAMA2* deletions (14/28, 50%) were predicted to induce frameshift truncations (out-of-frame deletions; Fig. 2), all in-frame deletions were found in MDC1A group in combination with a second truncating frameshift or a nonsense mutation, except in three patients with missense mutations instead. Nevertheless, these three patients (P4, P13, and P27) had typical MDC1A phenotypes. Strikingly, individuals with the same CNV may have different maximal motor milestones. For example, P13 carried the Chinese founder mutation, with a deletion of exon 4, but was independently ambulant, possibly due to compound heterozygosity with a missense mutation.

## Discussion

In this study, 34 cases from 29 families were genetically characterized in detail for pathogenic CNVs in *LAMA2*, following detection of non-recurrent genomic rearrangements among a large cohort of patients with *LAMA2* MD. Moreover, a modified NGS assay was developed to detect and identify *LAMA2* variants. Deletion of exon 4 was detected in eight non-consanguineous Chinese Han patients with MDC1A (10/37 disease alleles, accounting for 27% of all CNVs). Breakpoint analysis revealed AACAA microhomology at the junction in all 10 disease alleles, and haplotype analysis identified a founder mutation consisting of an in-frame deletion of 5,465 bp. Therefore, screening for *LAMA2* point mutations, followed by analysis of *LAMA2* CNVs, especially exon 4 deletion, may be appropriate as an initial strategy for patients with features consistent with congenital muscular dystrophy, such as muscular dystrophy combined with white matter changes in brain MRI.

Based on the two largest studies of *LAMA2* MD patients to date, we estimated that the overall frequency of CNVs may be as high as 18.6% (55/296 alleles), highlight the importance of screening for CNVs in suspected cases. These alleles consist of 37/192 alleles in our cohort, including from 96 patients with *LAMA2* mutations (Xiong *et al*.^7^ and this study), and of 18/104 alleles analyzed by Oliveira *et al*.^5^ in 52 patients. Our data also support the existence of two possible hotspots for large *LAMA2* deletions, one at exons 3 and 4 and another at exons 56 to 65 at the 3′ region of the gene, as first described by Oliveira *et al*.^5^. Strikingly, we detected 27 intragenic *LAMA2* deletions but only one intragenic duplication in our cohort. Indeed, only one other pathogenic heterozygous in-frame duplication of exons 28 and 29 has been documented previously^5^, and which is different from the novel duplication encompassing exons 5 to 8 in patient P16.

Currently, genomic sequencing is the main strategy to diagnose pathogenic *LAMA2* variants, with the highest sensitivity of approximately 80%. aCGH and MLPA are frequently used to detect CNVs as well, although these methods do not resolve breakpoint junctions and genomic orientation. For example, aCGH may generate spurious calls due to non-biological hybridization signals. Indeed, patient P7 was diagnosed by aCGH as carrying a small deletion, but mapping endpoints by specific PCR and sequencing instead identified the arrangement as a founder mutation involving exon 4. Similarly, samples from P23 produced significantly more calls than average, and mapping subsequently failed due to high levels of noise. On the other hand, data obtained by MLPA were limited as well. The probe mixes P391 and P392 contain one or more probes for all *LAMA2* exons except 18, 44, and 48, therefore, CNVs at these sites may have been undetectable. We note that the false-positive rate for MLPA has been determined previously, along with sequence alterations that may compromise probe affinity. Several algorithms have been developed to identify CNVs from depth of coverage. However, the newest whole-exome sequencing platforms detect only deletions of three exons or larger, while smaller events are not reliably detected^29,30^. Whole genome sequencing has also been used as a standalone assay to detect genetic variants^31^, but this approach cannot detect CNVs shorter than 1 kb and may miss some longer CNVs as well. Our results show that more than half of *LAMA2* intragenic CNVs (53.4%, 15/28) span just one exon, the smallest 56 bp, highlighting the sensitivity of our method for CNV analysis. Thus, our custom-designed NGS approach may delineate large genomic rearrangements in addition to sequence variations, with high accuracy and specificity as well as reasonable cost and practicality.

Although several mechanisms have been proposed to drive genomic rearrangements^28^, we found that replication-based mechanisms such as fork stalling and template switching/microhomology-mediated break-induced replication may account for most *LAMA2* intragenic CNVs. In this study, we identified blunt ends at two breakpoint junctions and random nucleotide insertions <5 bp at three breakpoints, both features consistent with “information scars”^32,33^ that are typically formed by replication-independent non-homologous end-joining. Twenty simple non-recurrent rearrangements showed microhomology at the breakpoint junctions, also indicating non-homologous end-joining or fork stalling and template switching/microhomology-mediated break-induced replication^34^. Additionally, serial replication stalling was likely in one case, further indicating that replication-based mechanisms contribute to *LAMA2* intragenic CNVs. Indeed, high levels of microhomology at breakpoint junctions indicate replication-based mechanisms^35,36^, the significance of which may have been previously underestimated.

Mammalian replicons span 75–150 kb on average, and human genes are 27 kb on average^37^. Accordingly, the large size of *LAMA2*, as well as a larger number of intragenic replication origins, may explain the high frequency of intragenic CNVs. Using RepeatMasker, we found that 40.7% (11/27) of breakpoint junctions contained one or more repeat elements, a frequency higher than the average percentage of repeat elements in the entire *LAMA2* gene. Additionally, LINE elements are most likely than SINE elements to mediate gene rearrangements in *LAMA2*. For example, the upstream breakpoints in P26–P28 are fragments of L3 (chr6: 129816155–129816735), resulting in deletion of exon 59–63 in three unrelated individuals with the same breakpoint. Recurrence of this deletion may be due to a founder effect, but further studies are needed to validate this hypothesis. Additionally, 4/11 instances of rearrangements are clustered at two LINE elements at introns 9 and 12, which are thus potential intragenic-rearrangement hotspots.

In summary, we provide for the first time a novel perspective on the spectrum of CNVs in *LAMA2*. In particular, we demonstrate that deletion of exon 4 is a founder mutation in Chinese Han population and the exon itself being a mutation hotspot. Moreover, we describe a novel NGS approach to detect and sequence CNV breakpoints. Our locus-centered analysis provides valuable insight into the molecular aetiology of *LAMA2* MD, and may help clinicians provide accurate and reliable genetic counseling, prenatal diagnosis, and gene therapy for those at risk.

### Web resources

The URLs for data presented herein are as follows:

**Table:** 

Leiden Open Variation Database	https://databases.lovd.nl/shared/variants/LAMA2
BWA	http://bio-bwa.sourceforge.net/
HaplotypeCaller of GATK	https://software.broadinstitute.org/gatk/
ANNOVAR	http://annovar.openbioinformatics.org/en/latest/
Polyphen-2	http://genetics.bwh.harvard.edu/pph2/
SIFT	http://si.jcvi.org/
Mutation Taster	http://www.mutationtaster.org
Oligo	http://www.oligo.net/downloads.html

## Electronic supplementary material

Supplementary material
