# Supplementary material for: Deletion of exon 4 in LAMA2 is the most frequent mutation in Chinese patients with laminin α2-related muscular dystrophy
Source: Sci Rep. 2018 Oct 9;8:14989. doi: 10.1038/s41598-018-33098-3 (PMC6177444; doi:10.1038/s41598-018-33098-3)
Supplement: Supplementary file 1 — Supplementary material [file 41598_2018_33098_MOESM1_ESM.pdf]

Supplementary Material

Deletion of exon 4 in *LAMA2* is the most frequent mutation in Chinese patients with laminin α2-related muscular dystrophy

Lin Ge[1,8], Aijie Liu[1,8], Kai Gao[1,8], Renqian Du[2], Juan Ding[1,8], Bing Mao[3], Ying Hua[4], Xiaoli Zhang[5], Dandan Tan[1,8], Haipo Yang[1,8], Xiaona Fu[1,8], Yanbin Fan[1,8], Ling Zhang[2], Shujuan Song[6], Jian Wu[7], Feng Zhang[2], Yuwu Jiang[1,8], Xiru Wu[1,8] & Hui Xiong[1,8]*

[1]Department of Pediatrics, Peking University First Hospital, Beijing 100034, China. [2]Obstetrics and Gynecology Hospital, Institute of Reproduction and Development, Fudan University, Shanghai 200011, China. [3]Department of Neurology, Wuhan Children's Hospital, Wuhan, Hubei 430015, China. [4]Department of Pediatrics, Wuxi Children's Hospital, Wuxi, Jiangsu 214023, China. [5]Department of Pediatrics, The Third Affiliated Hospital of Zhengzhou University, Zhengzhou, Henan 450052, China. [6]Department of Medical Genetics, School of Basic Medical Sciences, Peking University, Beijing 100191, China. [7]MyGenostics, Inc., Beijing 101318, China. [8]Beijing Key Laboratory of Molecular Diagnosis and Study on Pediatric Genetic Diseases, Beijing 100034, China.

*Correspondence and requests for materials should be addressed to H.X. (Department of Pediatrics, Peking University First Hospital, No. 1 Xi'an Men Street, West District, Beijing 100034, P. R. China. Tel: +86 10 83573238; Fax: +86 10 66530532; E-mail: xh_bjbj@163.com.)

Supplemental material

P1: *LAMA2* Exon1 deletion

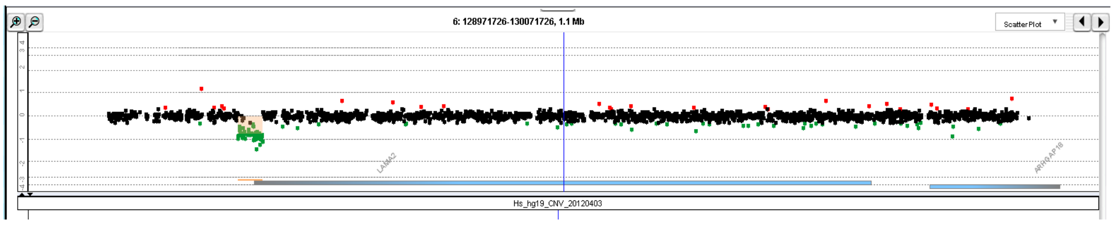

P3: *LAMA2* Exon2-3 deletion

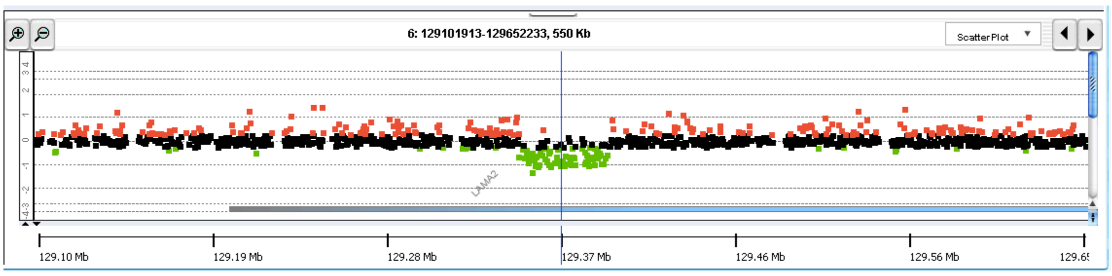

P4: *LAMA2* Exon2-9 deletion

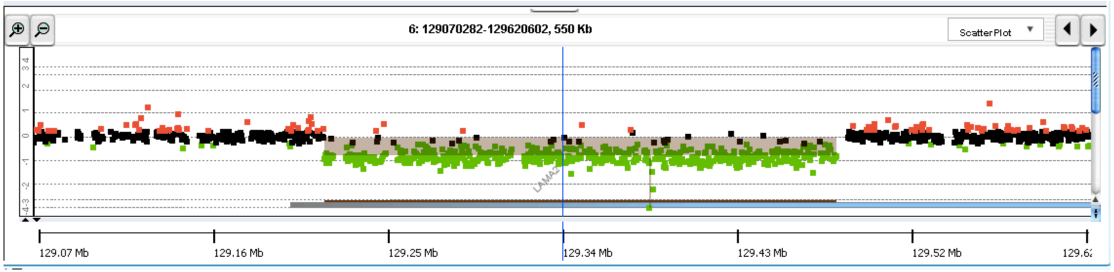

P6: *LAMA2* Exon3-4 deletion

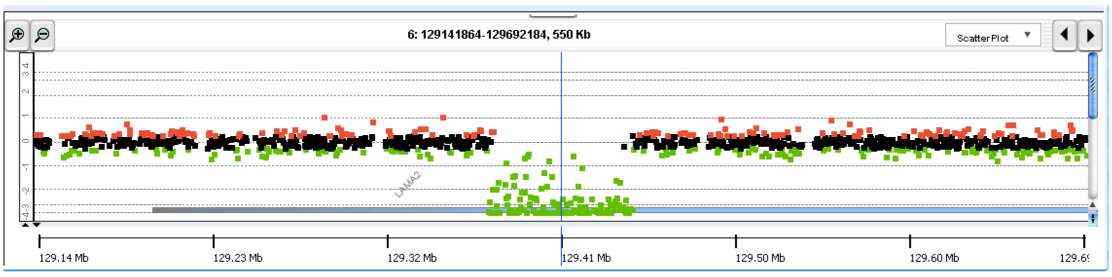

P7: *LAMA2* Exon4 deletion

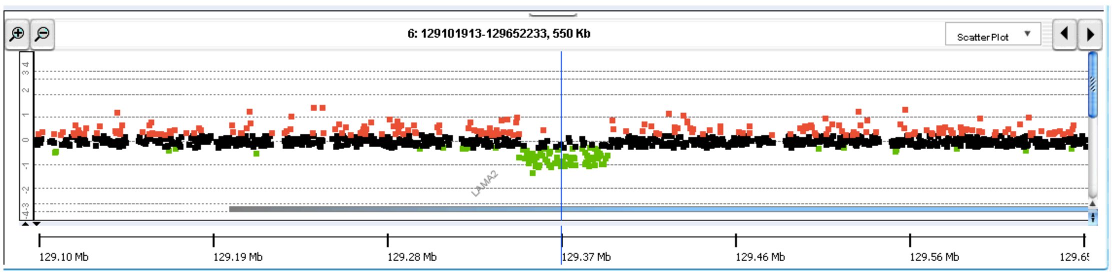

P8: *LAMA2* Exon4 deletion

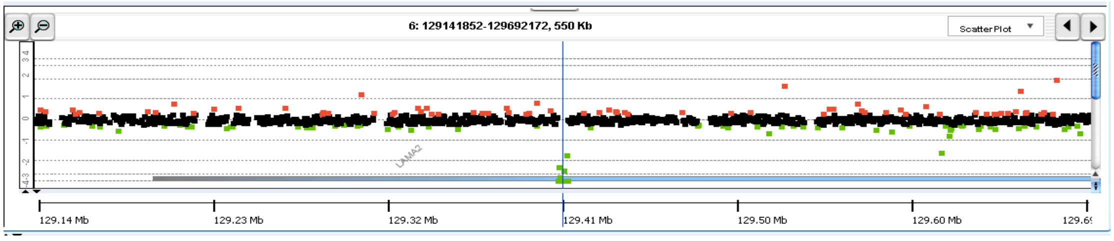

(continuing)

P9: *LAMA2* Exon4 deletion

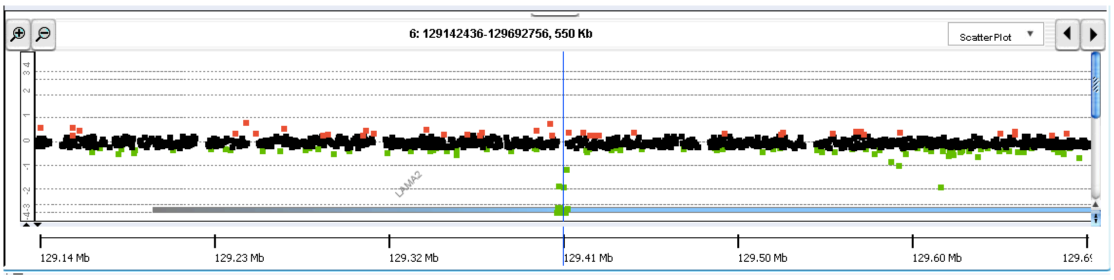

P10: *LAMA2* Exon4 deletion

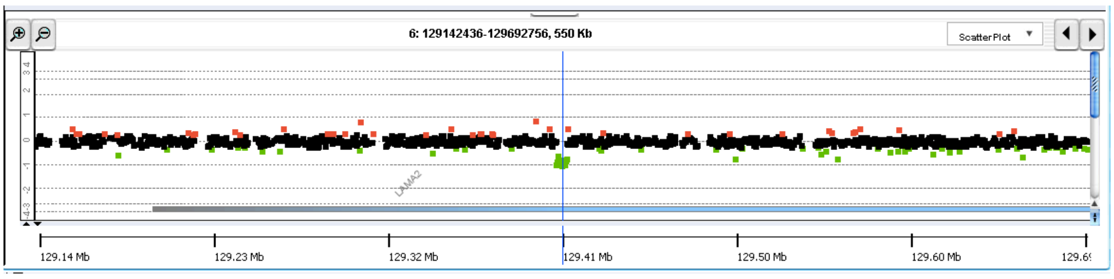

P11: *LAMA2* Exon4 deletion

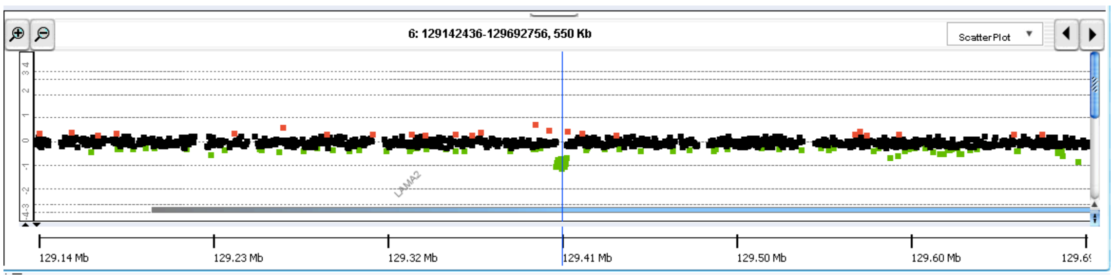

P15: *LAMA2* Exon5 deletion

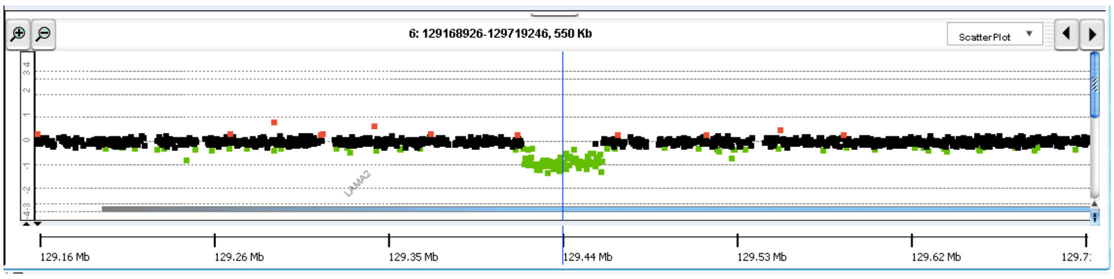

P17: *LAMA2* Exon10-12 deletion

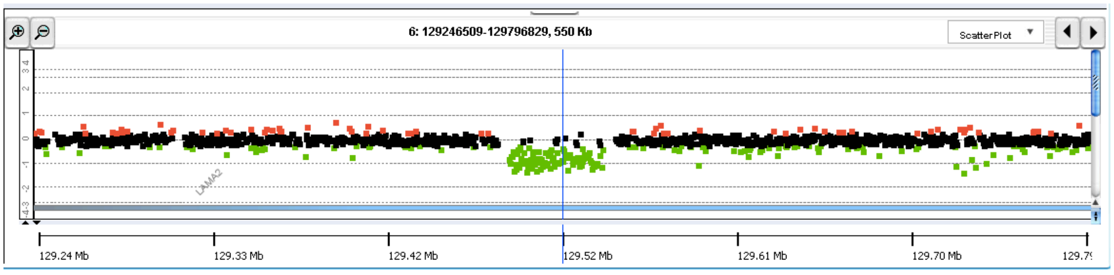

P21: *LAMA2* Exon36-65 deletion

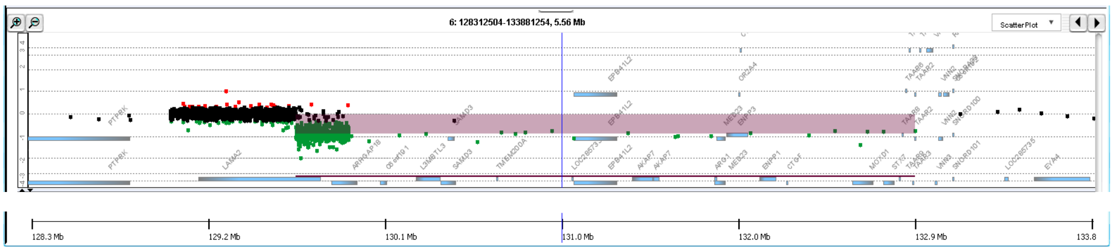

(continuing)

P22: *LAMA2* Exon41-47 deletion

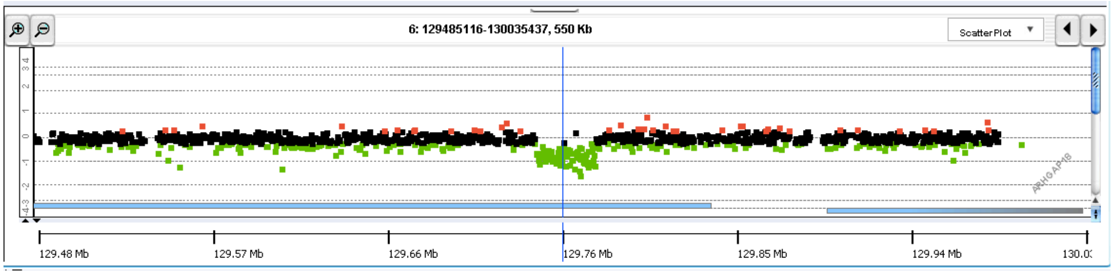

P23: *LAMA2* Exon49 deletion

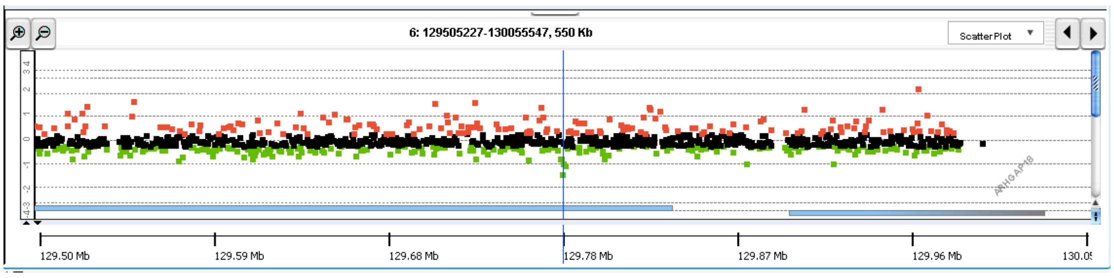

P26: *LAMA2* Exon59-63 deletion

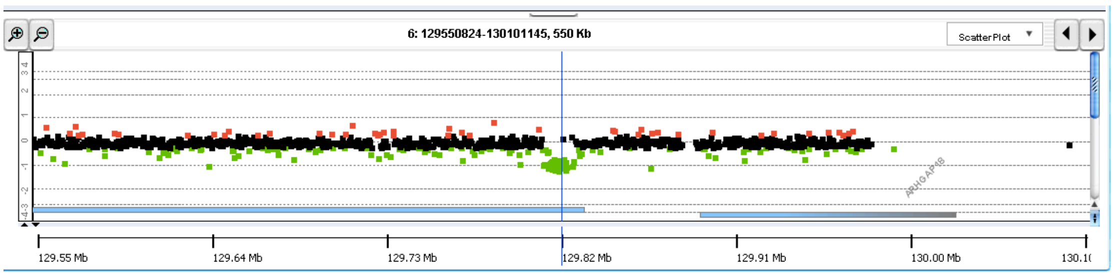

P29: No CNV detected

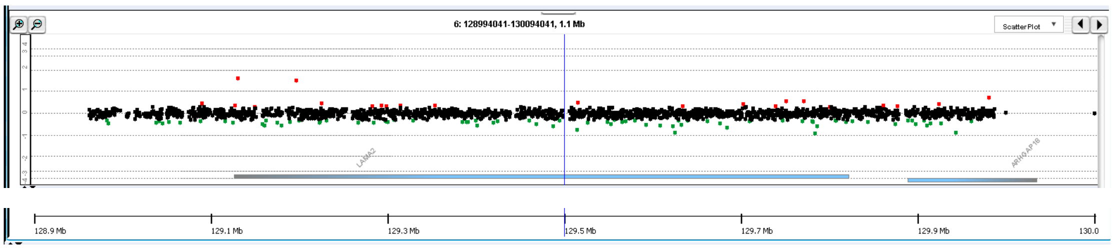

(end)

Figure S1. High-resolution aCGH results of 16 subjects with *LAMA2* MD.
The X-axis represents the chromosome location, while the Y-axis represents the normalized log2 Cy5(patient)/Cy3(healthy control) fluorescence intensity thresholds -1 (loss) and 1 (gain), respectively. Each point represents a oligonucleotide probe. Array CGH results revealed gains (in red) and/or losses (in green) of small chromosomal regions. Copy-number sizes were measured by the distance between the position of the first and last oligonucleotide probes included in the algorithmically determined region of aberration, respectively.

P2: *LAMA2* Exon1 deletion

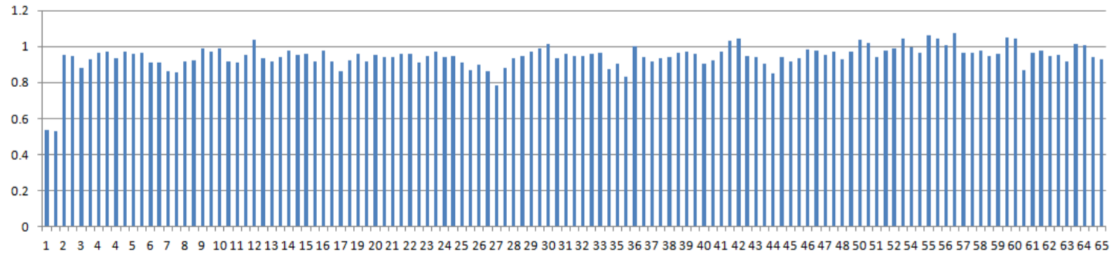

P5: *LAMA2* Exon2-12 deletion

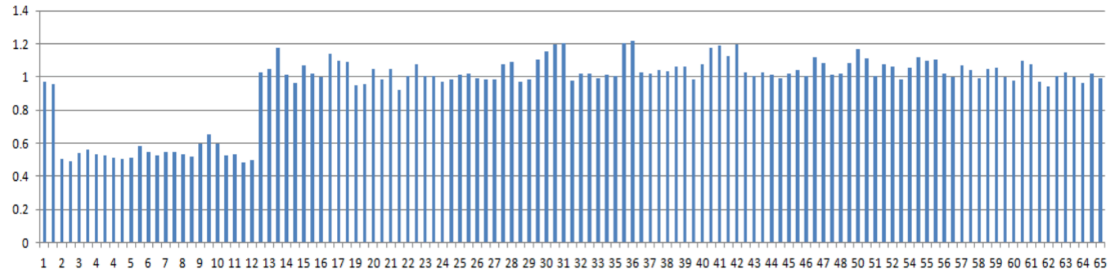

P16: *LAMA2* Exon5-8 duplication

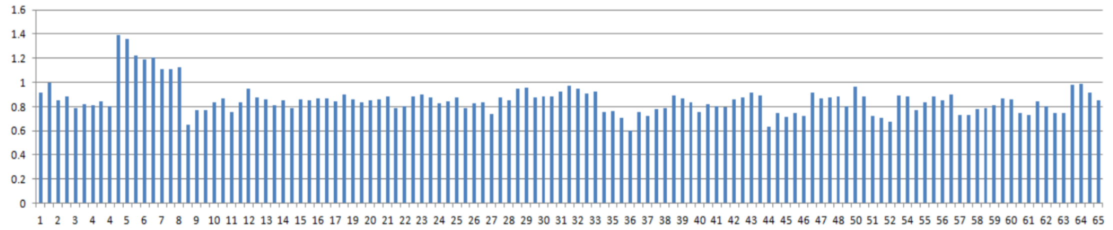

P17: *LAMA2* Exon10-12 deletion

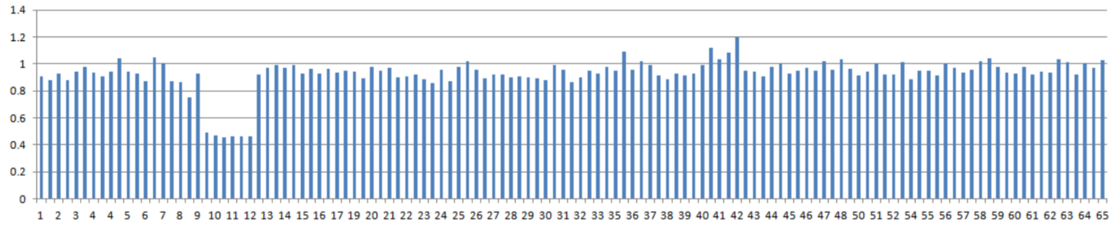

P18: *LAMA2* Exon13-14 deletion

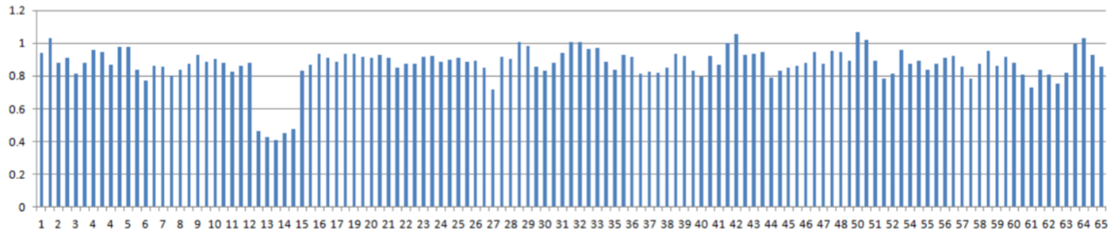

P19: *LAMA2* Exon20 deletion

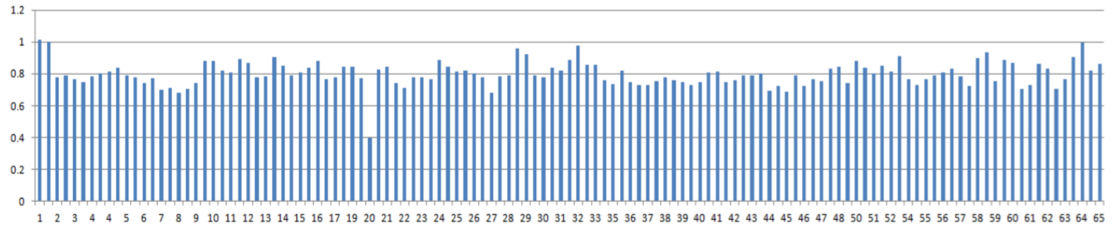

P20: *LAMA2* Exon30 deletion

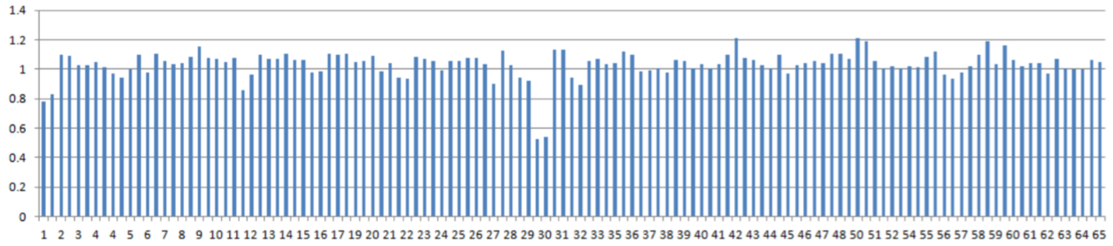

(continuing)

P21: *LAMA2* Exon36-65 deletion

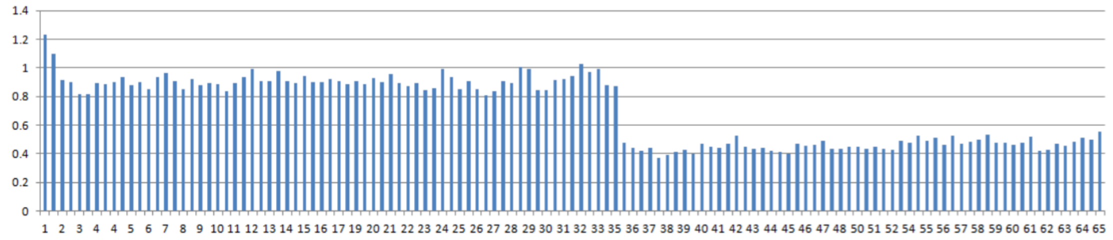

P23: *LAMA2* Exon49 deletion

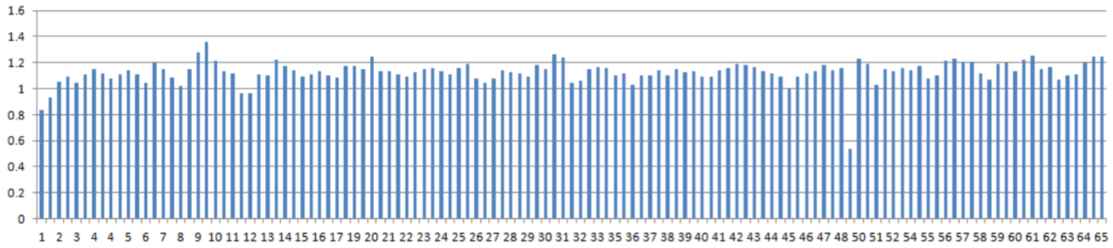

P24: *LAMA2* Exon49 deletion

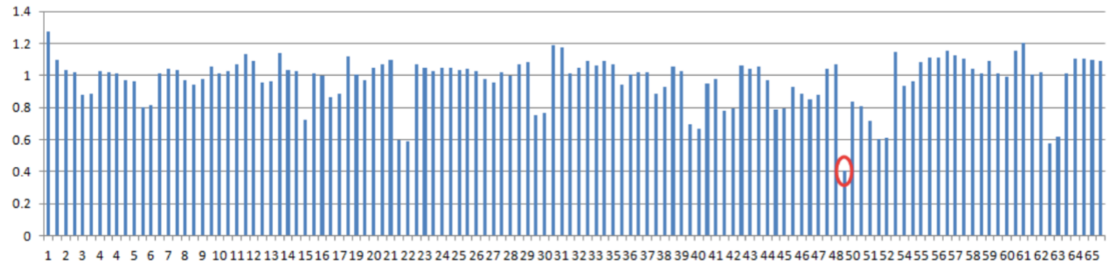

P25: *LAMA2* Exon49-57 deletion

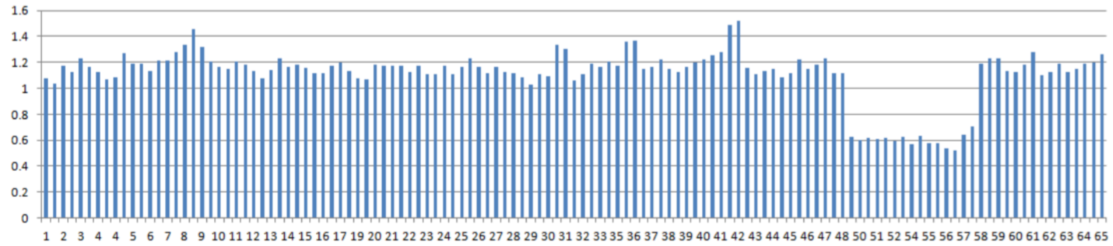

P27: *LAMA2* Exon59-63 deletion

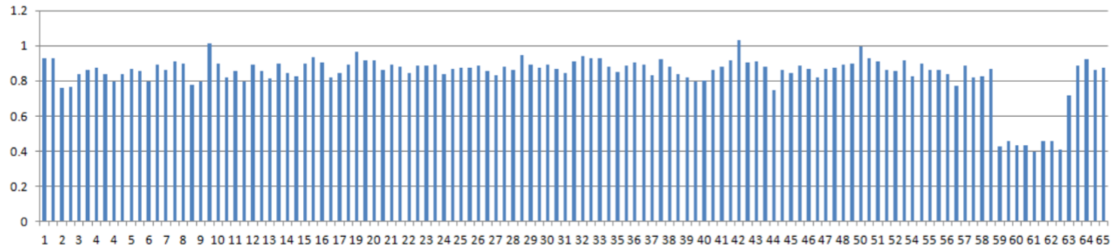

P28: *LAMA2* Exon59-63 deletion

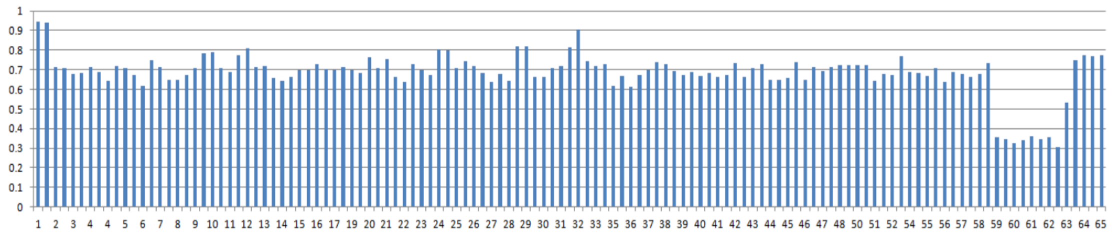

P29: No CNV detected

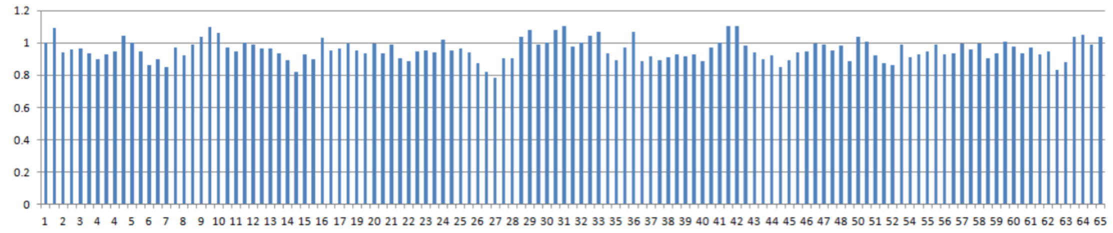

(end)

Figure S2. Next-generation sequencing results of 14 subjects with *LAMA2* MD.
The X-axis represents each *LAMA2* exon location, while the Y-axis represents the normalized ratio of sample reads to reference reads. Variants of copy number were speculated using the read-depth method. In brief, the ratio of sample reads to reference reads from control sample was calculated. Horizontal lines at 0.75 or 1.25 were used to highlight the thresholds using to call CNVs. The deficiency of CNV was defined, when the ratio was less than 0.75, while the amplification was defined, when the ratio was more than 1.25. The CNVs of whole *LAMA2* gene and exon regions were calculated respectively.

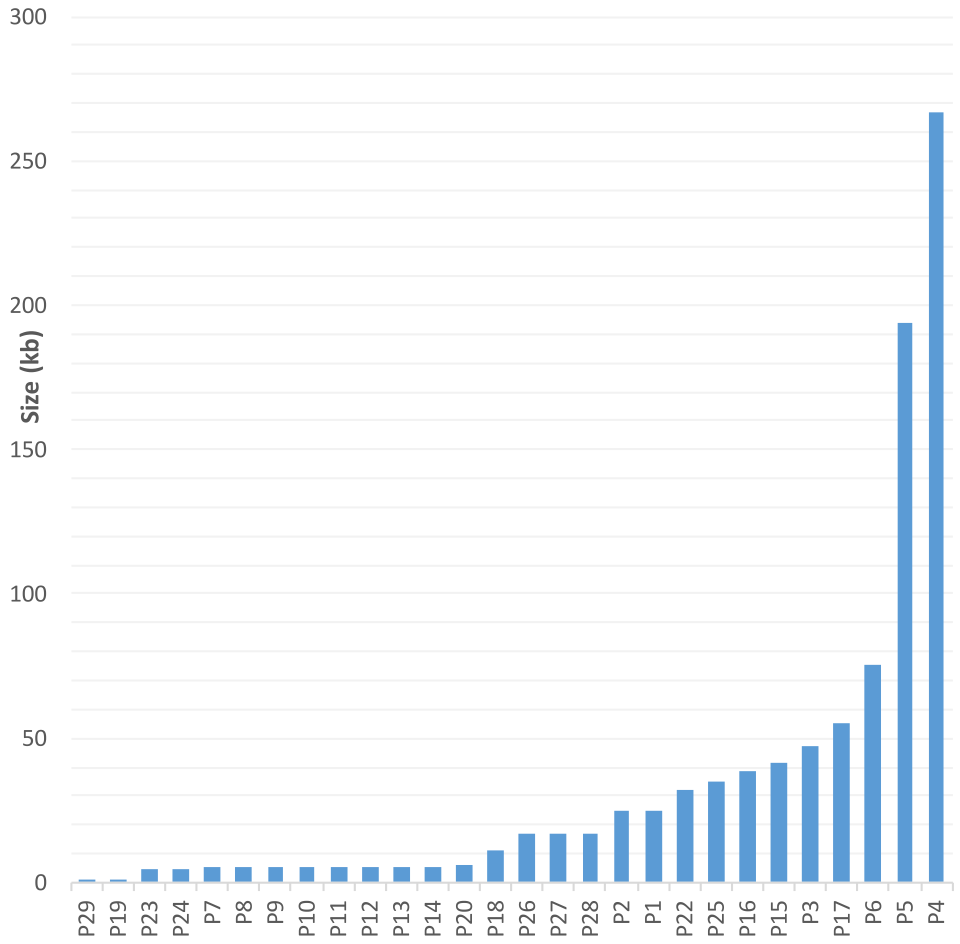

Figure S3. Size of *LAMA2* intragenic CNVs. *LAMA2* intragenic CNVs are plotted in ascending size order. Most *LAMA2* CNVs are small (median size is 14.3 kb).

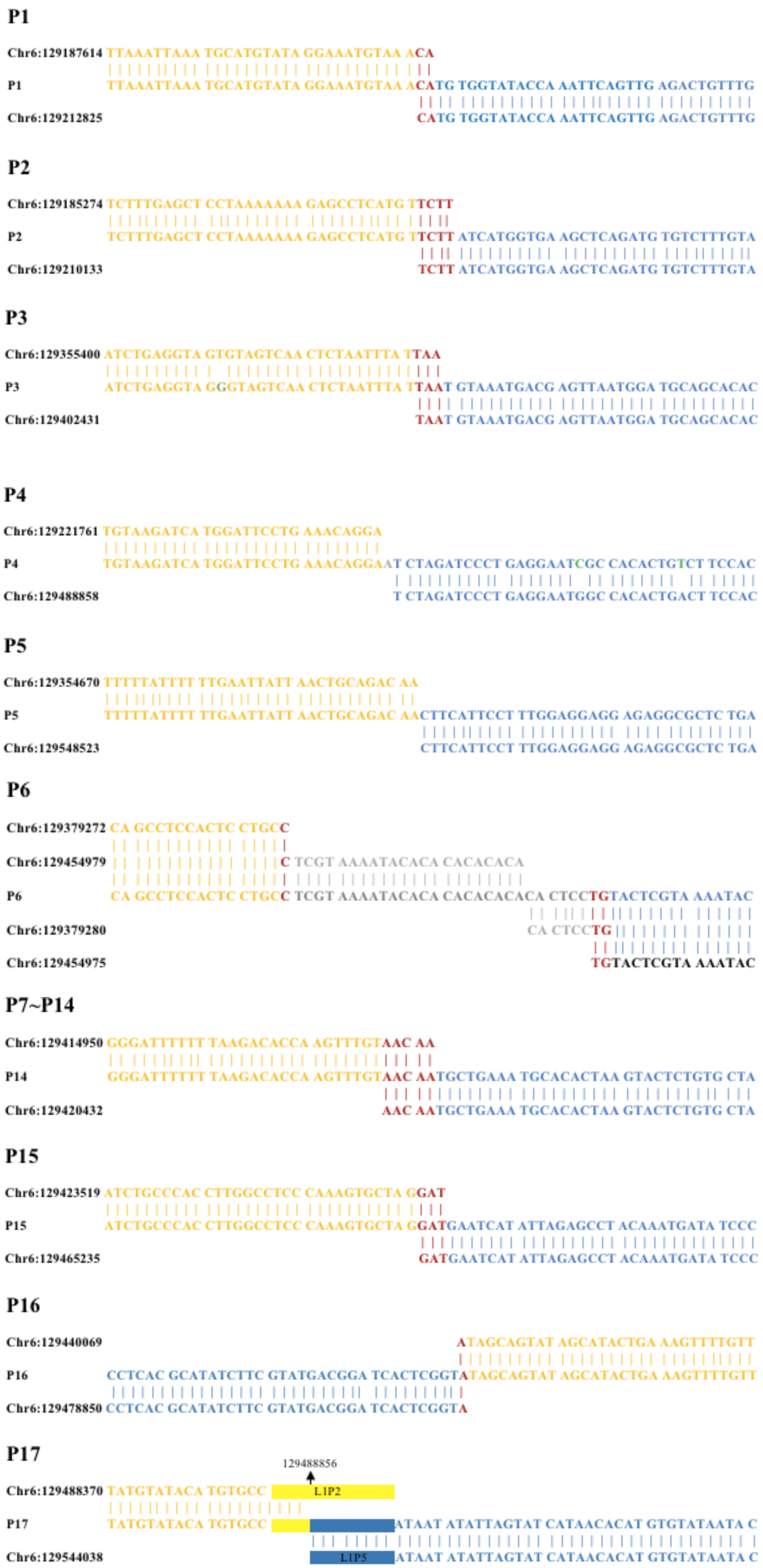
P1
Chr6:129187614 TTAAATTAAA TGCATGTATA GGAAATGTAA ACA
P1 TTAAATTAAA TGCATGTATA GGAAATGTAA ACATG TGGTATACCA AATTCAGTTG AGACTGTTTG
Chr6:129212825 CATG TGGTATACCA AATTCAGTTG AGACTGTTTG
P2
Chr6:129185274 TCTTTGAGCT CCTAAAAAAA GAGCCTCATG TTCTT
P2 TCTTTGAGCT CCTAAAAAAA GAGCCTCATG TTCTT ATCATGGTGA AGCTCAGATG TGTCTTTGTA
Chr6:129210133 TCTT ATCATGGTGA AGCTCAGATG TGTCTTTGTA
P3
Chr6:129355400 ATCTGAGGTA GTGTAGTCAA CTCTAATTTA TTAA
P3 ATCTGAGGTA GGGTAGTCAA CTCTAATTTA TTAAT GTAAATGACG AGTTAATGGA TGCAGCACAC
Chr6:129402431 TAAT GTAAATGACG AGTTAATGGA TGCAGCACAC
P4
Chr6:129221761 TGTAAGATCA TGGATTCCTG AAACAGGA
P4 TGTAAGATCA TGGATTCCTG AAACAGGAAT CTAGATCCCT GAGGAATCGC CACACTGTCT TCCAC
Chr6:129488858 T CTAGATCCCT GAGGAATGGC CACACTGACT TCCAC
P5
Chr6:129354670 TTTTTATTTT TTGAATTATT AACTGCAGAC AA
P5 TTTTTATTTT TTGAATTATT AACTGCAGAC AACTTCATTCCT TTGGAGGAGG AGAGGCGCTC TGA
Chr6:129548523 CTTCATTCCT TTGGAGGAGG AGAGGCGCTC TGA
P6
Chr6:129379272 CA GCCTCCACTC CTGCC
Chr6:129454979 C TCGT AAAATACACA CACACA
P6 CA GCCTCCACTC CTGCC TCGT AAAATACACA CACACACACA CTCCTGTACTCGTA AAATAC
Chr6:129379280 CA CTCCTG
Chr6:129454975 TGTACTCGTA AAATAC
P7~P14
Chr6:129414950 GGGATTTTTT TAAGACACCA AGTTTGTAAC AA
P14 GGGATTTTTT TAAGACACCA AGTTTGTAAC AATGCTGAAA TGCACACTAA GTACTCTGTG CTA
Chr6:129420432 AAC AATGCTGAAA TGCACACTAA GTACTCTGTG CTA
P15
Chr6:129423519 ATCTGCCCAC CTTGGCCTCC CAAAGTGCTA GGAT
P15 ATCTGCCCAC CTTGGCCTCC CAAAGTGCTA GGATGAATCAT ATTAGAGCCT ACAAATGATA TCCC
Chr6:129465235 GATGAATCAT ATTAGAGCCT ACAAATGATA TCCC
P16
Chr6:129440069 ATAGCAGTAT AGCATACTGA AAGTTTTGTT
P16 CCTCAC GCATATCTTC GTATGACGGA TCACTCGGTATAGCAGTAT AGCATACTGA AAGTTTTGTT
Chr6:129478850 CCTCAC GCATATCTTC GTATGACGGA TCACTCGGTA
P17
129488856
Chr6:129488370 TATGTATACA TGTGCC L1P2
P17 TATGTATACA TGTGCC ATAAT ATATTAGTAT CATAACACAT GTGTATAATA C
Chr6:129544038 L1P5 ATAAT ATATTAGTAT CATAACACAT GTGTATAATA C

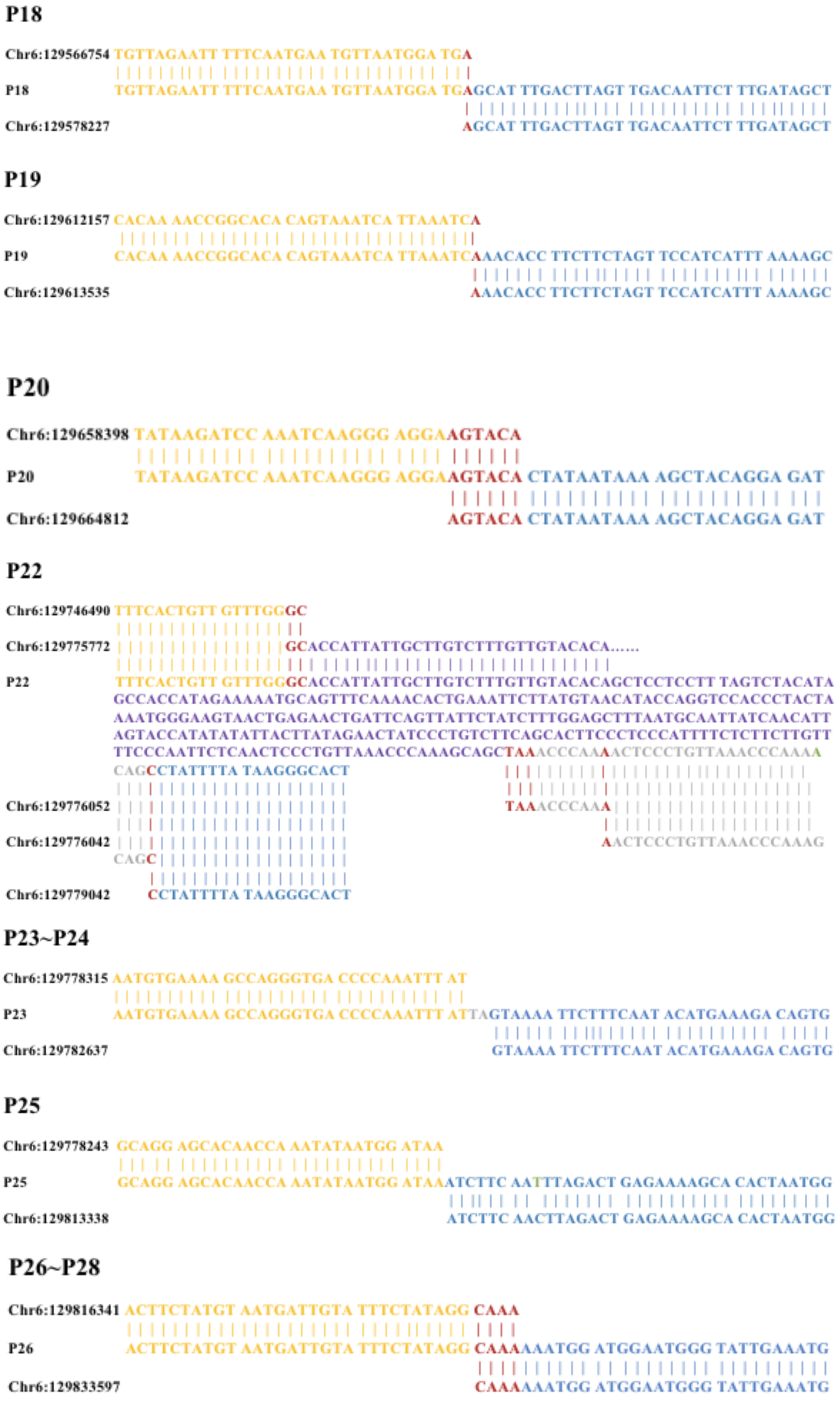

(End)

Figure S4. Junction fragment sequencing of *LAMA2* intragenic CNVs. The "upstream" (above) and "downstream" (below) reference sequences are aligned with sequence obtained from breakpoint PCR product (middle). The microhomology at the junction sites was annotated with red color, FoSTeS/MMBIR sequences were annotated with purple color, single- or oligo-nucleotide changes near breakpoints were annotated with green color, and inserted sequences between breakpoints were highlighted in gray color. The genomic coordinates were annotated next to the reference sequences GRCh37/hg19.

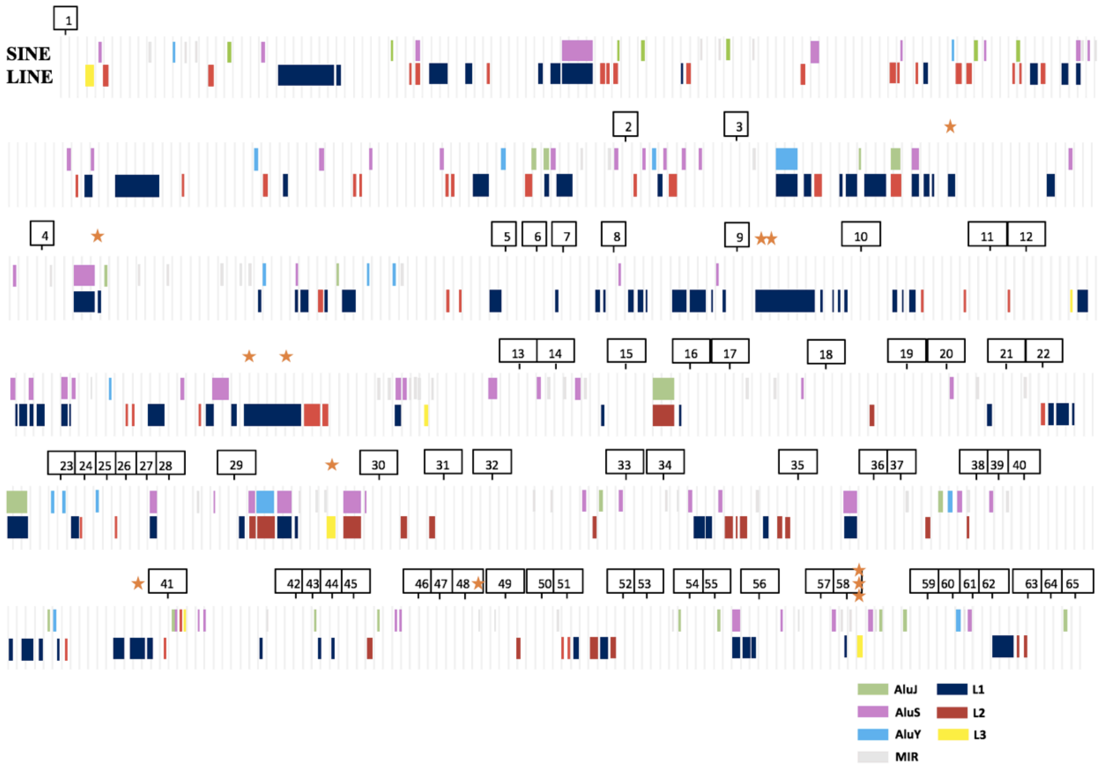

Figure S5. The Repetitive-Element Distribution within LAMA2. Overall, 96 *Alu* and 83 L1 elements > 100bp in size are present throughout the *LAMA2* non-coding region, and these highly homologous repetitive elements might mediate NAHR in various combinations. Exon numbers are shown in squares. The asterisks represent the likely NAHR events mediated by these repetitive elements and observed in this study. These *Alu* and L1 elements do not demonstrate equal recombination ability, none of *Alu* elements contribute to any recombination in this study. L1P2 (chr6: 129488385-129494343) and L1P5 (chr6: 129543585-129549735) have contributed LINE-LINE recombination

Table S1: Primers used to characterize the *LAMA2* CNVs

Subject	PCR Product (kb)	Primer	PCR Primer Position (Chr6/GRCh37/hg19)	PCR Primer Sequence 5'-3'
P1	～0.9	Forward	129187300-129187323	TAAGGGCACATAGTCTGTATCTCA
		Reverse	129213316-129213339	TAGTCTCTTAGGGTCTCCTCAAAG
P2	～6.9	Forward	129179401-129179425	AGGTTCACTGCTACAGATTAGTCCA
		Reverse	129211015-129211038	CGTGTCCAAATAAAATCCAAATAG
P3	～0.8	Forward	129354952-129354976	AGTCTATTTCTCTCCAACTGCCACC
		Reverse	129402738-129402762	AATCTTTCTCAAATCTTCCAGCACA
P4	～7.1	Forward	129220509-129220533	TGGCAGAGAACTAGAAGGGTATTTA
		Reverse	129494620-129494644	TGGTGAAGAATGTAGGGATGATAGT
P5	～2.0	Forward	129354335-129354359	GCCCTGAGGAACGATAATAGTTACT
		Reverse	129550068-129550092	CATAAGAGGAGCATCAGTCAATCAA
P6	～1.4	Forward	129378242-129378265	GCATTGAGGACTTAGTTTTGCTTT
		Reverse	129455281-129455305	GTGAGACGGAACAATCAGTACACTT
P7～14	～1.4	Forward	129413934-129413958	CTTCCATAGAGAGTGACCTTCATTA
		Reverse	129420804-129420828	TTTTCCCTAGAAGTGTTGAGAGATA
P15	～2.0	Forward	129422393-129422417	GAAAACCAGCAACAACTCTTATCTC
		Reverse	129466069-129466092	TAGAAGGTAGACAGGAGGAAGCAC
P16	～5.0	Forward	129475774-129475797	CACTGCTGGTATAAACTGCGAGAC
		Reverse	129441267-129441290	GGCTGGAATATCTTGAAATCTGCT
P17	～6.4	Forward	129488245-129488269	CCTATTTATCCCAGTCTTACTTCCA
		Reverse	129549827-129549851	GAACAGGAGTCTTTAGAGTTTGGTT
P18	～2.5	Forward	129565243-129565267	CGACACCTACAACTCTGGTTCATTT
		Reverse	129579148-129579172	GGACGGATGGGTAGATAGACAGATA
P19	～1.4	Forward	129611331-129611355	ACTCCAAGAAAAAGTGACTCCTAAA
		Reverse	129614037-129614060	CCTGATTTACAGACACATCTTCCT
P20	～2.1	Forward	129657273-129657296	CTGTGGAAAAAATAAACAGTGCTC
		Reverse	129665759-129665782	AACATTAGCCTGGGAAATCTTATC
P22	～0.9	Forward	129746435-129746459	TAGTCATCTCTGGTCATAGTCCCTC
		Reverse	129779575-129779599	CATATTCTGGTGTGGCAGTAGTCTT
P23～24	～1.7	Forward	129777476-129777500	GAAGAACTGGGAGAAATGGAACTAT
		Reverse	129783427-129783451	GCTATGTTGGTTTACTTTGGATTCA
P25	～2.6	Forward	129777654-129777678	CTCCTCCTGTTTATTTCATCTTTGT

		Reverse	129815265-129815289	TGTAGTGACTGTGACCATCTTTGTT
P26～28	～0.9	Forward	129815973-129815997	TCACCCTCATATACCTGAAAATCAT
		Reverse	129834033-129834057	CTTCTAAGAGATGCCTAGCCTGTTA
P29	～1.9	Forward	129832777-129832801	TGATACTCAGTTCAGTTGGTGTGGT
		Reverse	129834759-129834783	CATTAGTCAGTTTGGCAAGACATTC

Table S2. Quality control of next-generation sequencing results of 14 subjects with *LAMA2* MD.

Subject	Initial bases on target	Base covered on target	Coverage of target region	Effective bases on target	Average sequencing depth on target	Fraction of target covered with at least 4X	Fraction of target covered with at least 10X	Fraction of target covered with at least 20X	chip
**P2**	9351	9351	100.00%	8864214	947.94	100.00%	100.00%	100.00%	LAMA2_exon
**P2**	440494	431238	97.90%	176527125	400.75	96.47%	94.83%	92.51%	LAMA2
**P5**	9351	9351	100.00%	9444610	1010.01	100.00%	100.00%	100.00%	LAMA2_exon
**P5**	440494	430840	97.81%	189779201	430.83	96.25%	94.46%	92.11%	LAMA2
**P17**	9351	9351	100.00%	9256155	989.86	100.00%	100.00%	100.00%	LAMA2_exon
**P17**	440494	432194	98.12%	192712115	437.49	96.57%	95.32%	93.22%	LAMA2
**P18**	9351	9351	100.00%	9098833	973.03	100.00%	100.00%	100.00%	LAMA2_exon
**P18**	440494	429642	97.54%	177472354	402.89	95.84%	93.99%	91.33%	LAMA2
**P20**	9351	9351	100.00%	10343359	1106.12	100.00%	100.00%	100.00%	LAMA2_exon
**P20**	440494	430974	97.84%	212452691	482.31	96.37%	95.01%	92.83%	LAMA2
**P21**	9351	9351	100.00%	7251961	775.53	100.00%	100.00%	100.00%	LAMA2_exon
**P21**	440494	431634	97.99%	164570604	373.6	96.44%	94.96%	92.54%	LAMA2
**P25**	9351	9351	100.00%	10886809	1164.24	100.00%	100.00%	100.00%	LAMA2_exon
**P25**	440494	432057	98.08%	238270083	540.92	96.88%	95.91%	94.42%	LAMA2
**P27**	9351	9351	100.00%	8546247	913.94	100.00%	100.00%	100.00%	LAMA2_exon
**P27**	440494	431254	97.90%	173276987	393.37	96.40%	94.62%	92.35%	LAMA2
**P28**	9351	9351	100.00%	6981800	746.64	100.00%	100.00%	100.00%	LAMA2_exon
**P28**	440494	430297	97.69%	139077389	315.73	95.80%	93.76%	90.89%	LAMA2
**P29**	9351	9351	100.00%	9683154	1035.52	100.00%	100.00%	100.00%	LAMA2_exon
**P29**	440494	431123	97.87%	194115393	440.68	96.65%	95.21%	93.30%	LAMA2
**P24**	9351	9351	100.00%	9930144	1061.93	100.00%	100.00%	100.00%	LAMA2_exon
**P24**	440494	432005	98.07%	193655976	439.63	96.76%	95.36%	93.21%	LAMA2
**P23**	9351	9351	100.00%	11307700	1209.25	100.00%	100.00%	100.00%	LAMA2_exon
**P23**	440494	431853	98.04%	230970596	524.34	96.62%	95.39%	93.34%	LAMA2
**P19**	9351	9351	100.00%	8220763	879.13	100.00%	100.00%	100.00%	LAMA2_exon
**P19**	440494	429875	97.59%	160900770	365.27	95.89%	94.03%	91.13%	LAMA2
**P16**	9351	9351	100.00%	8843356	945.71	100.00%	100.00%	100.00%	LAMA2_exon
**P16**	440494	428891	97.37%	168930680	383.5	0.953	0.9316	0.9013	LAMA2
